# Cryostripping—A Safe and Efficient Alternative Procedure in Chronic Venous Disease Treatment

**DOI:** 10.3390/jcm11175028

**Published:** 2022-08-27

**Authors:** Sergiu-Ciprian Matei, Mervat Matei, Flavia Medana Anghel, Marius-Sorin Murariu, Sorin Olariu

**Affiliations:** 1 Department X of Surgery, “Victor Babeș” University of Medicine and Pharmacy Timișoara, Eftimie Murgu Sq. No. 2, 300041 Timișoara, Romania; 2 1st Surgical Clinic, “Pius Brînzeu” University Clinical Hospital Timișoara, Liviu Rebreanu Boulevard No. 156, 300723 Timișoara, Romania

**Keywords:** chronic venous disease, chronic venous insufficiency, cryostripping, minimally invasive venous surgery, endovenous procedures

## Abstract

Objective: The presentation of cryostripping as an alternative procedure useful in venous insufficiency treatment. Methods: This retrospective study presents the results of 1087 operated patients, including follow-ups. Cryostripping was practised in all mentioned cases. Patient follow-up was performed at one week, one month, and six months postoperatively by clinical examination, Doppler ultrasonography, CIVIQ-20 and r-VCSS questionnaires. Outcomes, complications, surgery and hospitalisation period, and benefits of the method were analysed. Results: Generally, good functional and aesthetic outcomes defined by clinical symptom remission, absence of insufficient veins on Doppler ultrasonography, QoL and r-VCSS improvement (*p* < 0.001) were obtained. Complications included bruising ⌀ < 2 cm (32.38%), haematoma (8.92%), saphenous nerve injury (3.49%), deep vein thrombosis (0.18%). Recurrence was noted in 2.94% cases. Mean duration of procedure was 42 ± 12.5 min, mean duration of hospitalisation was 1.05 ± 0.36 days. Compared to high ligation and conventional stripping, the postoperative complications were reduced; compared to other minimally invasive procedures, the costs were reduced. Conclusions: Cryostripping seems to combine the radicality and efficacy of the stripping technique with the cosmetic advantage of the endothermal procedures, being an effective therapeutic method perfectly adapted to the economic conditions of middle-income countries health system. It is also suitable as day-case surgery.

## 1. Introduction

Chronic venous disease (CVD) is a prevalent condition with global spread. The prevalence of CVD is highest in Western countries. More recent epidemiological studies of venous diseases in which the CEAP classification was used show a prevalence of 60–70% CEAP clinical class C0 and C1, about 25% for C2 and C3 and up to 5% for C4 to C6 with skin changes or venous ulcers. The incidence of varicose veins is approximately 2% per year [1,2]. Patients initially seek treatment to relieve symptoms of leg pain, discomfort, heaviness, and swelling, all of which impact their quality of life [1]. The modern surgical approach to varicose veins treatment is represented by venous reflux surgery. Whether dealing with a truncal or tributary reflux, it should be interrupted in order to prevent complications. The insufficient veins must be occluded or removed. A significant percentage of patients with CVD require surgical vein ablation as a therapeutic method [3]. Stripping operations and the less invasive endovenous thermal ablation have shown comparable results in saphenous vein insufficiency treatment [4]. However, endovenous ablation for CVD treatment is recommended over traditional surgery [5]. Surgical procedures have been diversified. In addition to classic stripping, endothermal and cryostripping methods have been developed.

Cryostripping is a minimally invasive surgical method used in varicose vein ablation, and is an effective and safe treatment modality in terms of postoperative complications, cosmetic results, and recurrence [6]. Curing varicose veins by cryostripping was practised for the first time in 1987, and since 1990 it has been successfully used in some phlebological centres in Germany. Although the technique of vein removal with a cryoprobe is simple, it is not widely practised [7]. Even though some studies have claimed that patients may prefer endovenous laser ablation because it is less painful and possesses low postoperative morbidity, in addition to offering a quicker return to normal activity [8], outpatient cryostripping is less costly and seems to be more effective [9]. The clinical outcomes of cryostripping are not inferior to those of endovenous laser ablation. Further, considering its cost effectiveness, cryostripping is a safe and feasible method for chronic venous disease treatment [10].

The objective of this study is to assess the efficacy and safety of this surgical technique, cryostripping, on the basis of an analysis of patient management, complications, clinical and economic implications.

## 2. Materials and Methods

This retrospective study analysed a series of parameters for patients suffering from CVD, which were operated in our Phlebology Department between September 2013 and September 2021. Cryostripping was introduced in the 1st Surgical Clinic, Phlebology Department of the “Pius Brînzeu” University Clinical Hospital Timișoara in September 2013. A total of 1250 patients were operated during the time period defined for this study. We included in the study group only those patients that came for clinical re-evaluations and for whom we had a follow-up of at least six months. A total of 1087 patients (1182 operated limbs) were admitted to the study (163 patients were not included due to the lack of or insufficient period of follow-up).

All patients were evaluated by duplex ultrasound before surgery. To highlight the venous reflux, we used a GE Healthcare Venue40 Doppler ultrasound, with a 12L-SC transducer, with all ultrasound examinations being performed by the same team of physicians. The “pulse-wave” Doppler examination showed the reflux at the ostium level in the cases of great saphenous vein (GSV) and small saphenous vein (SSV) by the Valsalva manoeuvre performed in the orthostatic position.

As a therapeutic intervention, cryostripping was practised in all those cases. Regional (spinal) or general (laryngeal mask) anaesthesia were preferred as the anaesthetic method, due to good intraoperative comfort and early postoperative mobilisation. The principle of cryostripping consists of venous catheterisation with a special probe that is cooled to −85 °C, causing it to adhere well to the vein, thus enabling its removal. In technical terms, the surgery begins by crossectomy. The ligation and section of the saphenous vein cross is practised at the entrance to the femoral vein. Distal vein catheterisation is performed then, retrograde from thigh to the lower leg in case of GSV and from the popliteal fossa to the malleolus, in the case of SSV. The probe is smooth and sustained by light calf flexion and extension movements on the thigh, and it can be easily inserted even if the saphenous vein path is tortuous. Connecting the probe to the ERBOKRYO device, cooling is carried out to −85 °C using liquid nitrogen. When the vein adheres to the probe, it is extracted by repeated traction at every 4–5 s, which is the time needed for the probe to cool. The complete removal of the insufficient vein takes about 45–60 s. When necessary, phlebectomies were additionally performed through skin punctures, using a Varady hook. In the case of small-calibre vessels, other sclerotherapy procedures were practised, to occlude them (especially polidocanol sclerotherapy). Compression stockings and phlebotonic medication were recommended in all cases after the procedure.

For the patients that underwent outpatient antiplatelet drug treatment for various other associated pathologies, we replaced this with low-molecular-weight heparins for one week prior to the elective surgery and for five days after it. The heparin dose was established on the basis of the cardiologist’s examination; dalteparin 5000 IU once per day, with subcutaneous injection being used in most of the cases.

Patients were evaluated by clinical examination and duplex ultrasound at seven days, one month, and six months postoperatively. The clinical examination evaluated the signs of remaining varicose veins, the presence of possible local complications, surgical wound healing, and specific CVD symptoms. Both residual healthy superficial veins and deep veins were evaluated by ultrasound. Quality of Life (QoL: Chronic Venous Insufficiency Questionnaire—CIVIQ-20) and revised Venous Clinical Severity Score (r-VCSS) were also evaluated before the procedure, and one month and six months after.

Outcomes (clinical signs, Doppler ultrasonography, QoL, r-VCSS), complications, surgery and hospitalisation period, associated postoperative drug treatment, and the benefits of the method from clinical and economical points of view were analysed.

All data were entered into a Microsoft Excel spreadsheet (Microsoft, Bellevue, WA, USA). Data are reported in the text as mean with two decimal places or mean ± standard deviation of the mean. For the statistical analysis we used the Data Analysis module and the statistical tests. We analysed the normality distribution of variables using the Kolmogorov–Smirnov test, and for the comparative statistical analysis we used Student’s t test. The Kaplan–Meyer method with the log-rank test (Mantel–Cox) was applied to evaluate the occurrence of recurrence. A *p* value threshold below 0.05 was considered for statistical significance.

## 3. Results

The study group comprised 756 female and 331 male patients. The age range was between 19 and 87 years old, the mean age being 48.53 ± 12.17 years (median 48). Mean body mass index (BMI) was 28.6 ± 4.43 (median 28.6), the range being 19.7–40.02. Regarding CEAP classification, the enrolled patients were in all stages of the disease in which venous reflux is encountered, as follows: 864 patients (79.48%) in C2 and C3 stages, 164 patients (15.08%) in C4 stage, 59 patients (5.42%) in C5 and C6 stages.

Venous reflux was found at GSV level in 958 cases, at SSV level in 91 cases, and both major superficial venous trunks (GSV and SSV) were insufficient in 38 cases.

**Figure 1:**
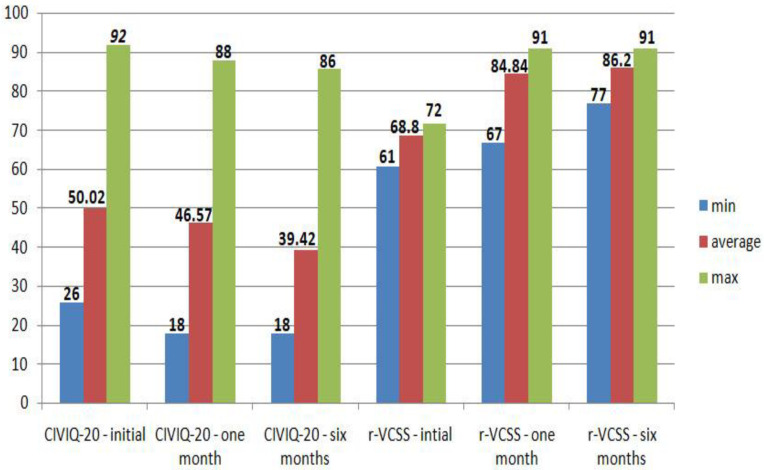
CIVIQ-20 and r-VCSS results at baseline, one month, and six months among the study group.

Clinical signs and symptoms of CVD (visible varicose veins, inflammation, pain, and oedema) improved significantly from the baseline at each follow-up in 1047 cases (96.32%), according to the clinical examination practised postoperatively by the physician. Absence of reflux or thrombosis with normal blood flow were noted in 1080 cases (99.35%). No cosmetic issues from remaining varicose veins, iatrogenic skin lesions or keloid scars were noticed. Analysing questionnaire results, highly significant statistical differences were noted for both CIVIQ-20 and r-VCSS when comparing the baseline with results at one month and six months (*p* < 0.001). However, the scores (one or both) were not improved in 28 cases (2.57%). Score results are presented in Figure 1.

Several short-term complications were noticed. Regarding short-term complications, we encountered bruising (⌀ < 2 cm) in 352 cases (32.38%), postoperative haematoma in 22 cases (2.02%), deep vein thrombosis (DVT) in two cases (0.18%) with onset on the fifth or sixth postoperative day, paraesthesia due to saphenous nerve injury remitted in less than six months (mean period 2.4 ± 0.9 months) in 37 cases (3.4%).

The average duration of the interventions was 42 ± 12.5 min, depending on the number of varicose collateral/perforating vessels. The time necessary for crossectomy, collaterals ligation and saphenous vein cryostripping varied between 15 and 35 min, surgery time being prolonged in cases of increased number of other varicose veins having to be occluded/removed.

Mobilisation after surgery was early, according to the practised anaesthesia (1–2 h for general and 3–5 h for regional). We did not encounter significant complications of anaesthesia: transitory urination disturbances in 25 cases (2.29%), nausea or vomiting in 41 cases (3.77%), transient arterial hypotension in two cases (0.18%). Although the possibility of performing the surgery under local anaesthesia +/− tumescent anaesthesia has also been described in literature, we did not practise this method, considering that it is not so comfortable for the patient, and the costs are not significantly reduced compared to the anaesthetic methods that we used.

Hospitalisation period was calculated in days (≤ 24 h—one day; 24–48 h—two days; 48–72 h—three days, etc.). The mean hospitalisation period was 1.05 ± 0.36 days (median 1, range 1–5), patients being discharged the same day or the next day after the surgery in most of the cases. The cases in which was necessary a longer than one day hospitalisation time were due a better control of the postoperative pain, or for the observation of other associated comorbidities or risks.

Regarding postoperative medication, the main aim was pain control and prevention of thrombotic events. We used nonsteroidal anti-inflammatory drugs such as metamizole or ketoprofen for a two-day mean period in 1066 cases with good results. Subsequently, the analgesic medication was adjusted and administered according to the affirmative pain symptomatology of each patient. In 21 cases (1.93%), other medication associations were necessary, as follows: metamizole/ibuprofen in 16 cases (1.47%), metamizole/acetaminophen in four cases (0.36%), metamizole/nefopam hydrochloride in one case (0.09%). After those two cases with complications of deep vein thrombosis, for patients at risk we considered low-molecular-weight heparins (subcutaneous injection) to be recommended for several days after the procedure; no blood clot complications were encountered then.

Regarding long-term complications, in one case (0.09%), we encountered paraesthesia due to saphenous nerve injury persisting after six months. We did not encounter other side effects, severe complications, or death. Recurrence (new varicosities that were evident at clinical examination or venous reflux present on ultrasound) was observed in 32 patients. Of these, five (0.45%) had recurrences at six months, eight (0.83%) at 12 months, and 19 (1.74%) at 18 months or more. The success rate of the cryostripping procedure seems to be higher than 97% in the early term and mid term, and about 92.14% after five years (we have a five-year follow-up only for 407 cases; a two-year follow-up for 928 cases, and a one-year follow-up for 1012 cases—unpublished data).

**Figure 2:**
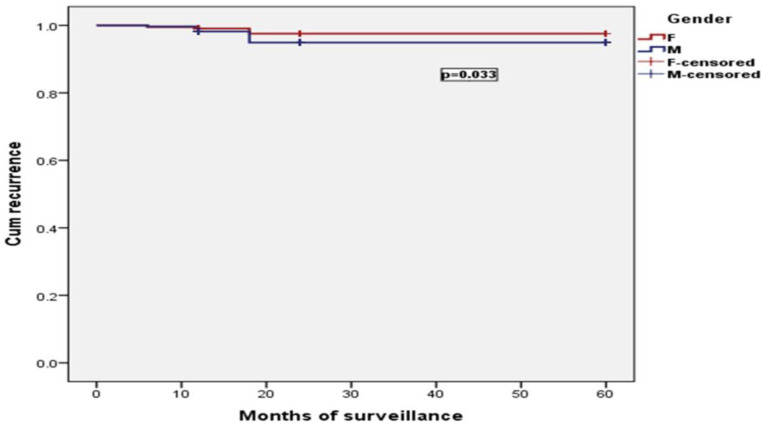
The frequency of recurrence was statistically significantly influenced by the gender of the patients (*p* < 0.001).

**Figure 3:**
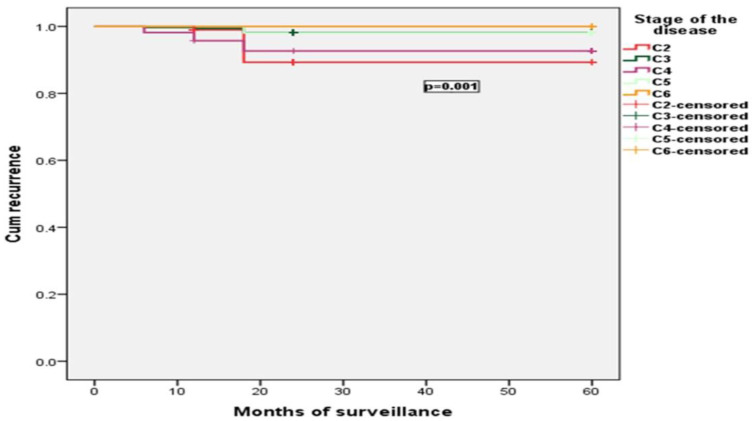
The frequency of recurrence was statistically significantly influenced by the CVD stage (*p* < 0.001).

The frequency of recurrence was statistically significantly influenced by the gender of the patients and CVD stage (*p* < 0.001). Obesity and age category did not influence recurrence *p* = 0.067, *p* = 0.982. Results are presented in Figure 2 and Figure 3.

The costs of the materials (liquid nitrogen, single-use supplies, medication, sutures, steri-strips, dressings) are around EUR 52 ± 10 per surgery. Considering the price of the cryo-device, probes (which can be re-sterilised and are reusable), hospitalisation and labour (surgical and anaesthesiological team), the cost of a surgery rises to about EUR 800 ± 250 per surgery.

## 4. Discussion

For many years, conventional stripping was considered the standard procedure for GSV insufficiency treatment, with this procedure nowadays being recommended only for a minority of patients with specific anatomical pathologies, as well as in countries with limited health and economical resources [10,11,12]. Despite this, because the concept of crossectomy and stripping has proved to be highly effective and has become the basic principle in varicose vein insufficiency surgery, all other minimally invasive therapeutic techniques that have been developed in the last decades have had to compete with crossectomy and stripping. In western industrialised countries, due their technical development, the classic stripping procedure has been replaced with highly effective, minimally invasive procedures [11]. Cryostripping, endovenous laser therapy (EVLT), radio-frequency ablation, VenaSeal and ultrasound-guided foam sclerotherapy have been developed. Among them, both cryostripping and laser therapy have been reported to be less traumatic, with lower rates of complications and recurrences when compared to conventional stripping [10]. Cryostripping respects all the principles of venous insufficiency surgery, bringing the additional advantage of a minimally invasive approach compared to the conventional procedure, such as the reduced dimensions of the groin fold incision because of the small diameter of the probe, the fact that there is no need for contralateral or other incisions because the freezing probe has a very strong adhesion, short execution time, and reduced complication rate [13]. A percutaneously guided probe approach has also been described [7], but we have not used this method.

Follow-up results were quantified by clinical examination, duplex ultrasound, and CIVIQ-20 and r-VCSS questionnaires. CIVIQ-20 and r-VCSS have been validated for the assessment of treatment effects for CVD patients in multinational studies, too [14,15]. Regarding our results, cryostripping is an effective procedure, with good outcomes generally being registered. However, several complications have been noticed. According to literature, complications after this procedure may vary from postoperative pain, skin pigmentation, bruising, haematoma, lymphatic complications (lymphoedema), cellulitis, superficial thrombophlebitis, wound infection, deep vein thrombosis, and cutaneous nerve damage with paraesthesia [6,16,17]. Postoperative pain and bruising are the major short-term side effects reported, and may appear after EVLT, as well [18]. We did not encounter cellulitis, superficial thrombophlebitis, wound infection, seroma or lymphoedema. Comparing our data to a previous study [6], bruising (32.38% vs. 64.9%) and haematomas (2.02% vs. 2.3%) were observed in a lower percentage. An explanation for this may be the conversion of antiplatelet drug treatment to heparins in the cases which undergone outpatient antiplatelet drug treatment. All those cases I nwhich subcutaneous haematic collections were developed were successfully treated conservatively or by needle aspiration; no additional incisions for drainage were necessary. We also used elastic bandages immediately after the surgery; after 24 h they were removed and replaced with compression stockings. When possible, compression (by elastic stockings or wraps) should be applied after surgical or thermal procedures [19], their role in complications prevention being acknowledged [20]. Superficial nerve injuries are very common during venous insufficiency classic surgery [21]. Even if this complication rate decreases greatly when minimally invasive procedures are used, nerve injury remains a risk with thermal ablation or cryostripping, too. In cases where it does occur, the injury tends to be transient [22]. We encountered this complication in a small percentage of cases (3.49%), with conservative treatment bringing good results in most of them. Regarding the other complications that we encountered, our results are similar to those found in the literature data [8,23,24]. Major adverse events like pulmonary embolisms could appear after minimally invasive procedures. The literature data describe this as a very rare complication (0.02%) that may occur after endovascular radiofrequency ablation [25]. We did not encounter any severe complications in our study group.

Because cryostripping is a radical procedure and the insufficient veins are removed, the recurrence risk by recanalisation is null. The causes of recurrence of varicose veins after various procedures are different, which has important implications for treatment [26]. Unlike endovenous procedures, where recurrence may happen due to recanalisation in most cases [10,27], recurrence after cryostripping is due to neovascular vessels, new varicose veins as a consequence of disease progression or residual/untreated veins. Comparing the procedure type, the frequency of recurrences is uncertain. While some studies conclude that, at a five-year follow-up, a significantly higher varicose vein recurrence rate appeared after EVLA compared to surgery [28,29,30], some papers claim that recurrence was comparable between those techniques within a period of three years and after five years [31,32], and no significant difference was determined regarding the recurrence rate in the comparison of radiofrequency ablation to surgery or endovenous laser therapy [33]. Comparing our data with respect to recurrence rate to another recent study presenting GSV stripping, the results are quite similar [34]. Comparing our data with other studies that present EVLT, the results regarding mid-term recurrence are quite similar, too, but there are differences between cryostripping and EVLT results regarding long-term recurrence. According to the literature data, occlusion rate in endovenous laser therapy was about 95–99.5% in the early term and mid term, and between 64% and 95% after five years [10,27,28,32]. From this point of view, cryostripping may be a more effective procedure.

Analysing the duration of the procedure, our results are comparable to those of other studies [6,16]. The procedure duration may vary regarding several aspects like the number of collateral veins which have to be treated, patient constitution (height, weight, body-mass index), and surgical team experience. Overall, cryostripping is not a time-consuming procedure. Because mobilisation after surgery is early, and recovery and social reintegration are fast, the intervention is perfectly suitable as a day-case surgery [35]. However, it should be noted that unlike other endovenous procedures, the patient needs to be monitored for at least a few hours after surgery due to the performed anaesthesia. This requires adequate space, equipment, and qualified medical personnel.

Skin burns are a potential complication of thermic endovenous procedures that may appear in a small percentage of cases [36]. Aesthetically, unlike endothermal procedures, the cryostripping avoids skin thermal injury which may provide a bad result. No skin lesions resulted from vein ablation when withdrawing the probe. The incision may be considered a disadvantage of this method from an aesthetic point of view, but we did not encounter keloid scars. Additionally, because the incision is placed in the groin fold or in the popliteal fossa, the scar is not evident.

Cost-effectiveness analyses of current varicose vein treatments have been described in various studies. The results differ, depending on several aspects. While some references suggest that EVLA is the most cost-effective therapeutically, and that it should be considered the treatment of choice for suitable patients [37,38], others convey that radiofrequency ablation is the treatment with the highest median rank for net benefit, with mechano-chemical ablation second, EVLA third, high-ligation surgery fourth, cyanoacrylate glue occlusion fifth, and conservative care and ultrasound-guided foam sclerotherapy sixth [39]. The main aspect influencing these analyses is the economic level of the countries where the studies were conducted, followed by clinical and economic evidence, characteristics of patients, disease or treatment, and contextual factors potentially affecting decision-making [40]. Although high-ligation and stripping procedures are not at the forefront of cost-effectiveness analysis, our results confirm the effectiveness of the cryostripping method and its low cost. Additionally, some studies conducted by Disselhoff revealed that EVLA and cryostripping were similarly effective in patients with varicose veins. In terms of cost per quality-adjusted life year gained, outpatient cryostripping appeared to be the dominant strategy, but endovenous laser yielded comparable outcomes for an additional cost [8,9]. One aspect that significantly reduces the cost of the intervention is the fact that the cryostripping probe is not disposable, unlike radiofrequency and laser fibres, which are disposable and have a higher cost. Comparing cryostripping to other procedures in practice like VenaSeal or EVLT, it is much cheaper than those, resulting in a lower complication rate than the classic stripping, and a higher satisfaction level from patients, too. In our country, intravenous procedures are not reimbursed by health insurance, and they are practised only in private clinics. The price ranges generally vary as follow: EVLT EUR 1300–1900; radio-frequency ablation EUR 1200–2000; VenaSeal EUR 1800. Many patients thus prefer hospitalisation. This aspect has made cryostripping a popular procedure in our healthcare system. This procedure is perfectly adapted to the economic conditions of the health system in middle-income countries, like Romania.

Considering the results of our study corroborated the discussed literature data, we can consider the following advantages of cryostripping: high success rate, reduced number of complications and recurrences, short execution time, the slightest postoperative pain, good aesthetic results, cost effectiveness, and feasibility as a day-case procedure. The main disadvantages of this procedure are the necessity for an incision, and spinal or general anaesthesia. It is appropriate to consider the specific indications and limitations of each of the techniques [41]. Even if some authors recommend endovenous ablation with laser or radiofrequency combined with phlebectomies before surgery or foam [42], we consider that cryostripping may be the indication of choice in venous insufficiency treatment, especially in the public health system. This is an effective procedure, and it may be indicated in all cases of valvular insufficiency requiring a saphenectomy (GSV or SSV), all cases with reflux on collaterals cross or trunks of saphenous veins, in patients with varicose veins and distal lower limb oedema, and in the case of venous leg ulcers.

## 5. Conclusions

Cryostripping is an effective therapeutic method that can be used under any circumstances. It combines the radicality and efficacy of the stripping technique with minimal invasiveness and the cosmetic advantage of endothermal procedures, leading to good functional and aesthetic outcomes. Among the advantages of this procedure are high success rate, reduced number of complications, short execution time and reduced costs, including the possibility of practicing this procedure as day-case surgery, too. Cryostripping should be considered among endovenous procedures for saphenous veins incompetence treatment, especially in middle income countries, due to its economic advantages.

## Data Availability

The data generated in this study may be requested from the corresponding author.
